# Institutional Needs

**Published:** 1913-03-22

**Authors:** 


### Institutional Needs.

### SANITARY TREATMENT OF FLOORS AND FURNITURE.

On the whole, perhaps, the wooden floor, for the ward at any rate, has gained the greatest amount of support from institutional authorities, and as a means of solving the problem of its sanitary treatment and general cleansing the "Ronuk" specialities were soon recognised as being germicidal but non-corrosive disinfectants, without the pronounced smell which usually attaches to such products. Hospital "Ronuk" Liquid and Hospital "Ronuk" Concentrated are in constant use in many institutions, and the latter can also be applied to furniture as well as flooring. If there is a problem equally important to that of cleansing the floors of wards and corridors it is that of combining polish with sanitation in hospital wooden furniture. It is not, however, always recognised that "Ronuk" is rather a basis than a result, so that, with modifications suited to different purposes, it is incorporated into various products for the cleansing and polishing of boots, harness, waterproofs, and metal, in addition to floors and furniture. Now that so many institutions have a contract with this firm for polishing their floors, it may be thought unnecessary to refer to the specially designed brushes and polishers which have been put on the market for the application of the "Ronuk" preparations. But there is always a certain amount of work which cannot be put out to contract, and for this, the less imposing in quantity, but equally important in a sanitary sense, the "Ronuk" preparations are to be recommended. If the base has been proved a success for the polishing and sanitary treatment of the ward floor, it is not likely to fail when incorporated in other forms for analogous purposes.

